# Ultraviolet Irradiation Effect on Apple Juice Bioactive Compounds during Shelf Storage

**DOI:** 10.3390/foods5010010

**Published:** 2016-02-18

**Authors:** Edmundo Juarez-Enriquez, Ivan Salmerón, Nestor Gutierrez-Mendez, Enrique Ortega-Rivas

**Affiliations:** 1 Centro de Investigación en Alimentación y Desarrollo, Av. Rio Conchos S/N, Parque Industrial, Cd. Cuauhtémoc, Chihuahua 31570, Mexico; edjuen@gmail.com; 2 Graduate Program in Food Science and Technology, The Graduate School, Autonomous University of Chihuahua, Chihuahua 31125, Mexico; isalmeron@uach.mx (I.S.); ngutierrez@uach.mx (N.G.-M.)

**Keywords:** ultraviolet irradiation, non-thermal food preservation, bioactive compounds stability, apple juice storage

## Abstract

Clarified and standardized apple juice was ultraviolet-irradiated to inactivate polyphenol oxidase enzyme and microbiota, and its effect on bioactive compounds and stability during storage was also evaluated. Apple juice was irradiated with 345.6 J/cm^2^ and treatment effect was evaluated in terms of color, antioxidant capacity, polyphenol content, pH, titratable acidity and total soluble solids. Using a linear regression design, inactivation kinetic of polyphenol oxidase enzyme was also described. In addition, a repeated measures design was carried out to evaluate apple juice during 24 days of storage at 4 °C and 20 °C. After irradiation, reduction of antioxidant capacity was observed while during storage, ascorbic acid content decreased up to 40% and total polyphenol content remain stable. Ultraviolet irradiation achieved a complete inactivation of polyphenol oxidase enzyme and microbiota, keeping apple juice antioxidants during ultraviolet treatment and storage available until juice consumption. UV-treated apple juice can be used as a regular beverage, ensuring antioxidant intake.

## 1. Introduction

A frequent consumption of apples has been correlated with healthy life, due to their antioxidant capacity and beneficial phytochemical compounds. Whole apple consumption promotes a LDL-cholesterol reduction [1], and polyphenols from apple juice help to reduce body fat [2]. Especially apple polyphenols have been linked with anticarcinogenic effects [3] and, most recently, referred to the prevention of colorectal cancer in man [4].

The presence and bioavailability of those beneficial compounds in processed apple juice are mainly related to the extraction and further preservation process [5]. Ultraviolet (UV) treatment has been used as an alternative preservation method for thermal treatment [6] to preserve those biochemical compounds.

Donahue *et al.* [7] and Basaran *et al.* [8] showed the UV treatment effectivity for pasteurization of cloudy and clear apple juice using *E. coli* as a target, reducing a minimum of five log cycles with 20 and 14 mJ/cm^2^ irradiation, respectively. Later Caminity *et al.* [9] observed a negligible effect of UV dose on phenol content; however, a decrease in antioxidant capacity after treatment was noted. The aim of the present study was to investigate the effect of UV treatment on clarified apple juice and its evolution during 24 days of storage.

## 2. Experimental Section

### 2.1. Raw Materials

Golden delicious apples were donated by a local farmer and juice was extracted in a pilot scale vertical press. Ascorbic and citric acid (800 mg/L and 325 mg/L) were added to the juice prior to clarification process to prevent enzymatic oxidation and color changes [10]. Clarification was achieved with an enzymatic mixture of glucoamylase, pectin metil esterase, pectinlyase and polygalacturonase enzymes donated by ENMEX S.A de C.V. (DF, Tlalnepantla, Mexico) for 12 h at 4 °C.

The reagents sodium carbonate, l-ascorbic acid, 2,6-dichloroindophenol, 2,2-diphenyl-1-picrylhydrazyl and gallic acid where provided by Sigma (St Louis MO, USA). Metaphosphoric acid and methanol where obtained from Fisher (Pittsburgh PA, USA) and Folin-Ciocalteu solution was from MP Biomedicals (Solon OH, USA).

### 2.2. Juice Characterization Colorimetric Measurements

Titratable acidity, pH and soluble solids (°Brix) were determined by AOAC 942.1.15, AOAC 942.1.04 and AOAC 932.12 methods respectively. Soluble solids were evaluated as °Brix at 20 °C using an ABBE-3L Refractometer (Milton Roy Inc., Rochester, NY, USA). The pH was measured by direct reading at 20 °C in an Orion Benchtop pH/ISE meter Model 420A (Orion Research Inc., Boston, MA, USA). Acidity was measured by titrating with 0.1 N NaOH to a pH end-point of 8.2, the result being expressed as mg malic acid/mL of sample.

Ascorbic acid, Trolox Equivalent Antioxidant Capacity (TEAC) and total polyphenols content were determined according to the methodology reported by Juarez-Enriquez *et al.* [11]. The absorbance of the solutions was measured on a Pekin Elmer Lamda 25 (Waltham, Massachusetts, USA) and was correlated with its respective standard curve.

### 2.3. Colorimetric Measurements

Color components L, a, and b, based on the Hunter system, were measured in 15 mL of juice using a hand-hold Tristimulus Colorimeter Konica Minolta CR-410 (NJ, USA) in a dark room with white background [12]. The mentioned components were transformed to hue (H*) and chroma (C*) indices [13,14] by:

(1) $$H^{*}=\text{arctan}\frac{b}{a}$$

(2) $$C^{*}=\sqrt{a^{2}+b^{2}}$$

### 2.4. Polyphenol oxidase (PPO) Activity Determination

PPO activity was assayed using the method proposed by Cano *et al.* [15] with a modification. Aliquots of 0.2 mL of the clarified juice and 2.8 mL of a solution of 0.15 mol/L catechol in 0.05 mol/L sodium phosphate buffer (pH 6.5) were hand-mixed in a 3 mL plastic cell. The kinetic reaction was measured at 420 nm and 25 °C with the spectrophotometer. An increase of one absorbance unit in 10 min was defined as one enzyme unit.

### 2.5. UV Treatment and Kinetic Data

UV treatments were performed with an irradiation 0.032 W/cm^2^ at 25 °C in a Trojan UV MAX C4 (Ontario, Canada) with capacity of 3000 mL and enabled to work in batch mode. In order to obtain the inactivation kinetic data, aliquots of 5 mL were extracted every 10 min for 2 h and kept refrigerated at 4 °C until PPO activity determinations.

### 2.6. Statistical Analysis

All experiments were done in triplicate and significance level was set at 0.05. Data analysis was carried out with Minitab 16 (Minitab Inc., State College, PA, USA) using a General Linear Model (GLM) with Tukey test for data and means analysis. A linear regression design was performed in Minitab 16 to describe the inactivation kinetic of PPO enzyme. An additional repeated measures design was carried out with IBM SPSS v20 Software (IBM, New York, NY, USA) to analyze the effect of the storage time (24 days) in clarified apple juice processed with the calculated UV radiation for 100% PPO denaturation, over quality variables on juice stored at 4 °C and 20 °C.

## 3. Results

Table 1 shows all differences in physicochemical properties of thermal- and UV-clarified juice treatments and untreated juice. Brix degrees, acidity, color saturation, total polyphenol content and total ascorbic acid content do not present significant (*p* > 0.05) changes in UV-treated juice when compared with non-treated clarified juice. In this sense, pH, acidity, Brix degrees, total polyphenol content and color saturation were stable with no significant (*p* > 0.05) changes in thermal-treated juice. On the other hand, significant (*p* < 0.05) changes after UV treatment on TEAC and color expressed as hue were observed.

**Figure 1:**
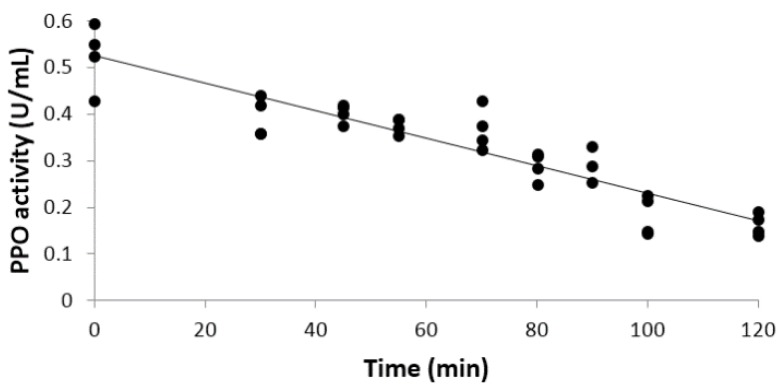
PPO deactivation kinetic by UV irradiation.

Complete PPO enzyme inactivation was achieved after 180 min, following a linear kinetic inactivation as described in Figure 1. The regression model was highly correlated (coefficient of determination *R^2^* = 0.84), and can be represented by the following relationship:

(3) $$Y=-0.003X_{1}+0.5257$$

where *Y* is the PPO activity and *X*_1_ is the treatment time with an irradiation of 0.032 J/cm^2^. The total UV dose received by apple juice was 345.6 J/cm^2^.

The physicochemical changes during the storage time of the UV-treated apple juice are shown in Table 2. Acidity, chroma, ascorbic acid content, total polyphenol content and TEAC parameters presented significant variation during the 24 days of storage at 4 °C and 20 °C. On the contrary, color change expressed as hue not was significantly (*p* > 0.05) affected during all storage time.

The UV-treated juice stored at 4 °C was also significantly (*p* < 0.05) affected on pH and Brix degrees parameters. However, the juice stored at 20 °C remains stable, and pH and Brix degrees remain without significant (*p* > 0.05) changes up to 24 days.

The effect of storage temperature on the physiochemical parameters is shown in Table 3. The unique parameter affected by storage temperature was the ascorbic acid content (*p* < 0.05); therefore, the storage temperature does not significantly affect (*p* > 0.05) pH, acidity, Brix degrees, total polyphenol content, antioxidant capacity and color.

UV-treated juice does not present microbial growth of molds or mesophilic bacteria after treatment and nor during storage time observations.

## 4. Discussion

The total UV dose applied to the apple juice was completely effective to decrease the native microbiota below detection levels. Previously, UV effectiveness was proved for some pathogenic microorganisms. Gabriel *et al.* [16] determined the UV decimal reduction dose for *Salmonella spp, E. coli* and *Listeria monocytogenes* in clear apple juice. They found *L. monocytogenes* strains where up to three-fold more resistant than other bacteria. In another study, Gachovska *et al.* [17] achieved a 3.46 log CFU/mL of *E. coli* after UV treatment for 2.3 s. Later, Caminiti *et al.* [9] treated apple juice with 0.177 W/cm^2^, and up to 53.10 J/cm^2^. Their minimum treatment of 2.66 J/cm^2^ achieved a complete reduction of *E. coli* and *L. inocua* from their samples.

In addition, PPO inactivation was completely achieved after UV treatment. Enzymatic activity reduction prevents antioxidant degradation and its inactivation by UV treatment was also previously reported. Falguera *et al.* [18] obtained a complete inactivation of *A. bisporus* polyphenol oxidase after UV treatment of 35 min at 0.28 W/cm^2^. However, native apple PPO is more resistant, as reported by Falguera *et al.* [19]. These researchers analyzed the PPO inactivation of four varieties of apples: Golden, Starking, Fuji and David. In all samples of centrifuged juice, PPO was inactive after 100 min at 0.28 W/cm^2^ of UV treatment.

Acidity, pH and Brix degrees were stable after UV irradiation, as reported previously. Caminiti *et al.* [9] found UV treatments at any dose did not affect pH, Brix degrees or phenol content, but a decrease in antioxidant capacity was observed after treatment. However, Azhuvalappil *et al.* [20] and Tandon *et al.* [21] reported significant changes (*p* < 0.05) in pH, Brix degrees and acidity in apple cider stored at 4 °C and 7 °C, after UV irradiation with 13 and 14 mJ/cm^2^, respectively, but these changes were attributed to microbial spoilage growth and not to UV treatment.

Differences in hue and antioxidant capacity after UV treatment are associated with double bounds disruption and oxidation reactions. These are promoted by UV irradiation and further photon absorption by double bounds or oxygen [22]. Antioxidant capacity of natural apple juice is mainly attributed to phenol antioxidants (87%) and ascorbic acid (6%) [23], while Tikekar *et al.* [24] described a decrease in ascorbic acid content during UV irradiation, up to 0.7–1.0 mg/min at 0.03 J/cm^2^. However, no significant changes (*p* > 0.05) in ascorbic acid content after UV treatment were observed, due to the protective effect of phenols [25] and pigments [18,19]. Diminution in TEAC by UV treatment effect was probably due to non-phenol antioxidant oxidation.

During storage, TEAC reduction due to ascorbic acid oxidation was observed, since polyphenol content remained with minimal variations. Ascorbic acid content decreased significantly faster in juice stored at 4 °C when compared with juice stored at 20 °C, but remained present for all storage time. However, contrary behavior was observed in polyphenol content, which decreases faster in samples stored at 20 °C than in those stored at 4 °C. This could be attributed to the different antioxidant activity of polyphenols and ascorbic acid at different temperatures. Polyphenols and ascorbic acid present higher antioxidant activity at ambient temperatures than lower temperatures [24,26]. In this sense, ascorbic acid can be the main antioxidant source at 4 °C and be degraded first and conversely at 20 °C, and polyphenols could be more reactive as an antioxidant source than ascorbic acid. Other authors also reported temperature-dependent degradation rates for ascorbic acid during storage. Marti *et al.* [27] observed a complete oxidation of ascorbic acid in pomegranate juice after four days of storage at 5 °C and 25 °C, but nevertheless, at 25 °C, ascorbic acid decreases 25% faster than that stored at 5 °C. Also, they reported that in a model system, ascorbic acid remained present up to 30 days at 5 °C.

Antioxidants also prevent color changes [28]. Hue variation during UV treatment was referred to oxidation reactions during treatment. On storage, hue does not present significant changes, while chroma decreased significantly, probably due to pigment oxidation or to a partial reactivation (enzyme resistant fraction) of apple PPO as observed by Juarez-Enriquez *et al.* [11].

## 5. Conclusions

UV treatment of apple juice offers an alternative to achieving a complete reduction of PPO activity and microbiota, becoming an attractive option to pasteurize apple juice in the food industry. After apple juice irradiation with ultraviolet light, polyphenols and ascorbic acid remain in the juice up to 24 days of storage at 20 °C. This implies UV-treated apple juice can be stored at ambient temperatures, ensuring antioxidant consumption.

## Figures and Tables

**Table 1:** Changes in physicochemical properties after UV treatment.

Property	Clarified Juice	UV Treatment
pH	3.74 ± 0.01 ^a^	3.54 ± 0.03 ^b^
Malic acid eq. (mg/mL)	6.19 ± 0.04 ^a^	6.06 ± 0.04 ^a^
°Brix	17.97 ± 0.06 ^a^	18.05 ± 0.07 ^a^
Ascorbic acid (ppm)	119.22 ± 5.04 ^a^	121.16 ± 0.75 ^a^
TEAC (mM)	10.99 ± 0.16 ^a^	9.85 ± 0.96 ^c^
Gallic acid eq. (mg/L)	343.17 ± 1.02 ^a^	346.02 ± 5.04 ^a^
Hue	81.37 ± 0.59 ^a^	87.57 ± 0.51 ^b^
Chroma	3.29 ± 1.21 ^a^	5.10 ± 1.32 ^a^

* Any two means in the same row followed by same letter are not significantly different (*p* > 0.05) by analysis of variance.

**Table 2:** Evolution of physiochemical parameters on UV-treated apple juice for 24 days storage at 4 °C and 20 °C.

Days	Temp (°C)	pH	Acidity (mg/mL)	°Brix	Hue	Chroma	Ascorbic Acid (ppm)	Gallic Acid Eq. (mg/L)	TEAC (mM)
1	4 °C	3.54 ± 0.03 ^a^	6.056 ± 0.039 ^a^	18.15 ± 0.07 ^ab^	87.57 ± 0.52 ^a^	5.10 ± 1.32 ^a^	121.16 ± 0.75 ^a^	347.70 ± 4.58 ^a^	9.884 ± 0.079 ^a^
6	4 °C	3.50 ± 0.03 ^a,b^	6.213 ± 0.077 ^a^	18.25 ± 0.07 ^a^	87.59 ± 0.41 ^a^	4.18 ± 0.64 ^a,b^	108.72 ± 0.83 ^b^	334.35 ± 1.11 ^b^	8.031 ± 0.144 ^b^
12	4 °C	3.48 ± 0.02 ^b^	6.168 ± 0.005 ^a^	17.25 ± 0.07 ^c^	88.01 ± 0.52 ^a^	3.32 ± 0.37 ^a,b^	105.60 ± 0.62 ^b,c^	332.35 ± 1.59 ^b^	7.445 ± 0.571 ^c^
18	4 °C	3.46 ± 0.02 ^b^	6.663 ± 0.131 ^b^	17.85 ± 0.21 ^a,b^	87.55 ± 0.95 ^a^	3.98 ± 0.14 ^a,b^	102.25 ± 5.48 ^c^	318.06 ± 1.97 ^b^	7.154 ± 0.250 ^c^
24	4 °C	3.44 ± 0.02 ^b^	7.084 ± 0.140 ^c^	17.70 ± 0.14 ^bc^	85.74 ± 1.41 ^a^	3.11 ± 0.13 ^b^	74.42 ± 2.32 ^d^	337.86 ± 5.06 ^c^	6.780 ± 0.121 ^c^
1	20 °C	3.54 ± 0.03 ^a^	6.056 ± 0.039 ^a^	18.05 ± 0.07 ^a^	87.57 ± 0.52 ^a^	5.10 ± 1.32 ^a^	121.16 ± 0.75 ^a^	347.70 ± 4.58 ^a^	9.884 ± 0.079 ^a^
6	20 °C	3.50 ± 0.03 ^a^	6.213 ± 0.077 ^a^	17.95 ± 0.07 ^a^	86.72 ± 0.45 ^a^	3.68 ± 0.52 ^a,b^	109.27 ± 0.64 ^b^	330.21 ± 1.11 ^b^	8.031 ± 0.144 ^b^
12	20 °C	3.51 ± 0.03 ^a^	7.017 ± 0.205 ^b^	17.70 ± 0.14 ^a^	88.02 ± 0.85 ^a^	2.91 ± 0.37 ^b^	106.14 ± 0.93 ^c^	310.09 ± 11.92 ^c^	7.751 ± 0.187 ^b^
18	20 °C	3.53 ± 0.02 ^a^	6.927 ± 0.353 ^b^	17.60 ± 1.14 ^a^	82.27 ± 2.80 ^b^	2.81 ± 0.34 ^b^	104.44 ± 0.09 ^d^	315.13 ± 3.31 ^b,c^	7.980 ± 0.081 ^b^
24	20 °C	3.54 ± 0.01 ^a^	7.375 ± 0.201 ^b^	17.65 ± 0.21 ^a^	86.40 ± 1.32 ^a^	2.59 ± 0.14 ^b^	96.07 ± 1.93 ^e^	330.57 ± 1.54 ^b^	6.876 ± 0.029 ^c^

* Two means in the same column followed by same letter are not significantly different (*p* > 0.05) by analysis of variance for each temperature.

**Table 3:** Temperature effect during storage on quality parameters of UV-treated apple juice.

Parameter	*p*
pH	0.928
Malic acid eq.	0.978
°Brix	0.123
Ascorbic acid	<0.001
Gallic acid eq.	0.882
Antioxidant capacity	0.953
Chroma	0.714
Hue	0.362

* Significant (*p* < 0.05) effect of temperature of storage by analysis of variance.
